# Key Issues at the Forefront of Diagnosis and Testing for Antiphospholipid Syndrome

**DOI:** 10.1177/10760296241306751

**Published:** 2024-12-18

**Authors:** Jesse Qiao, Jenique Bailly, Jessica Opie

**Affiliations:** 1 Department of Pathology and Laboratory Medicine, 14447 University of California, Irvine, USA; 2 Division of Haematology, Department of Pathology, 63726 University of Cape Town and National Health Laboratory Service, Groote Schuur Hospital, Cape Town, South Africa

**Keywords:** antiphospholipid syndrome, lupus anticoagulant, thrombosis, antiphospholipid antibodies, ISTH

## Abstract

Antiphospholipid syndrome (APS) is an autoimmune disorder characterized by antiphospholipid antibodies associated with thrombosis and pregnancy complications. Catastrophic APS is a severe form involving multiple organ systems with a high mortality rate. The pathogenesis involves antiphospholipid antibodies which target phospholipid-binding proteins and damage endothelial cells thus activating coagulation, triggering a pro-thrombotic state. Laboratory tests for antiphospholipid antibody detection include lupus anticoagulant testing in the coagulation laboratory and serological detection of anticardiolipin and anti-beta 2 glycoprotein I antibodies. Despite recent updates in the diagnostic criteria for APS the recent decades and our improved knowledge of this disease, there remain several key issues pertaining to diagnosis and testing with potential implications to patient management. Here we briefly review APS pathophysiology, strengths and weaknesses of classification criteria, laboratory challenges leading to test interpretation, and clinical management of this complex condition.

## Introduction

Antiphospholipid Syndrome (APS) is rare autoimmune disorder characterized by antiphospholipid antibodies (aPL), prolonged phospholipid-dependent coagulation tests and an increased risk of thrombosis and/or pregnancy-related complications.^1^ APS is often associated with autoimmune diseases particularly systemic lupus erythematosus (SLE) and is more common in females.^2^ The incidence is rare, varying from 1 to 50 cases per 100 000 per year and is associated with significantly increased mortality compared to the general population.^3^ Since its discovery and recognition in the twentieth century, APS has been known to present clinically with arterial or venous thrombosis and/or obstetric complications such as pregnancy losses and pre-eclampsia.^4,5^ Other clinical features may include livedo reticularis, thrombocytopenia, hemolytic anemia, valvular heart disease, and nephropathy.^6^ Catastrophic APS (CAPS) is a particularly rare and severe form of APS characterized by acute clotting of multiple vascular beds with multiorgan failure and a high mortality.^7^ The diagnosis and management of APS may be challenging due to disease heterogeneity and due to that fact that transient aPL may occur in a number of common settings particularly infection. In addition, aPL are found in a proportion of healthy people.^4,8^ aPLs can be demonstrated by lupus anticoagulant (LAC) testing in the coagulation laboratory or by immunological assays detecting antibodies to various proteins, most commonly cardiolipin (aCL) and beta-2-glycoprotein I (a-β2GPI).

While there has been significant progress in the development of diagnostic and classification criteria for APS,^5^ several clinical and laboratory challenges persist that will be discussed in this narrative review. In view of improvements in our understanding of APS pathophysiology and treatment, we aim to review and compare recent updates to the clinical and laboratory diagnostic guidelines for APS and summarize current best practices to aid clinicians and pathologists.

This review will also approach aspects that are not covered by the guidelines, such as the classification of CAPS and complexities of APS testing in patients on anticoagulation therapy. Furthermore, we review challenges in LAC testing such as the optimal method to determine of normalized ratios, caveats of the mixing study, seronegative APS, and false negative aPLs. Furthermore, we suggest possible avenues for improvement in APS laboratory testing and potential areas of research.

## Pathophysiology of Antiphospholipid Syndrome

**Figure 1.**
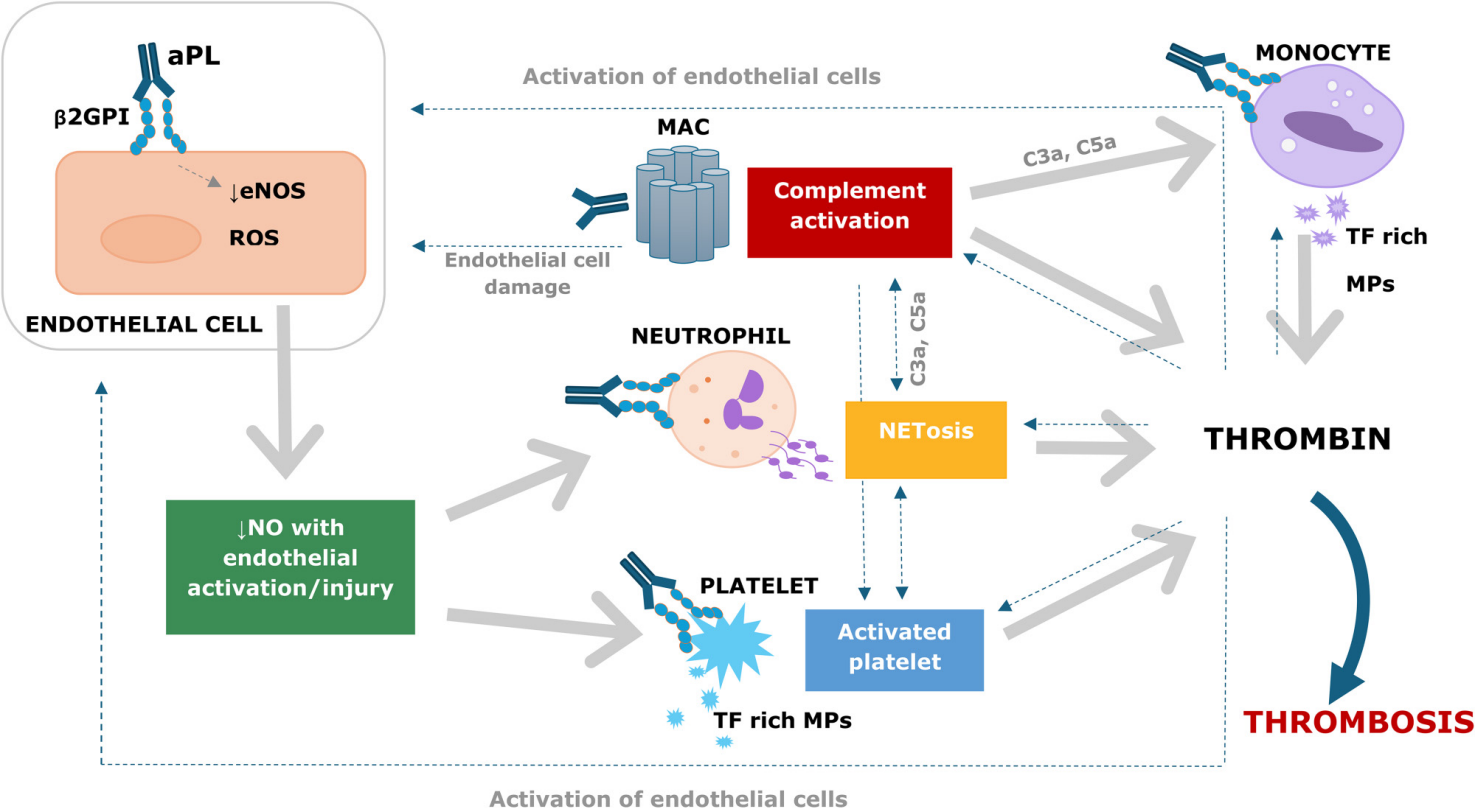
The pathophysiology of antiphospholipid syndrome.^8–12^

The presence and persistence of aPL is central to the pathogenesis of APS. The primary targets of APL are phospholipid-binding proteins, typically cardiolipin with β2GPI acting as a co-factor. Physiologically, β2GPI is known to regulate hemostasis and complement.^9,10^ Figure 1 summarizes the pathophysiology of APS at the cellular level. Endothelial cells are proposed to play a central role in pathogenesis, with endothelial cell dysfunction and circulating aPL being essential initiating factors.^11^ When β2GPI is exposed to anionic phospholipids due to platelet activation and inflammatory responses, it adopts an open conformation and exposes antigenic sites, including a cryptic antigen in domain I which binds autoantibodies. Another mechanism is that oxidative stress can unfold the β2GPI protein on endothelial cells, exposing these antigenic sites. Autoantibodies lock β2GPI in an open conformation and facilitate the binding of β2GPI:aPL complexes to receptors on endothelial cells, placental trophoblasts, monocytes, platelets, and neutrophils, thereby activating them.^11^ Complement activation then occurs with C3a and C5a generation and the formation of the membrane attack complex which results in endothelial cell injury and further activation of neutrophils, monocytes, platelets, and endothelial cells, as well as activation of coagulation.^8,9,12^

Antiphospholipid antibodies (aPL) are denoted within the top left box and bind to beta-2 glycoprotein I leading to reduced nitric oxide production and endothelial cell activation/injury. NETosis leads to platelet activation and complement activation which stimulate thrombin formation. aPL mediates the activation of complement via immune complex formation. Complement activation leads to endothelial cell injury and formation of C3a and C5a, and further endothelial cell activation, NETosis, platelet activation and monocyte activation with microparticle release (ref). Additionally, platelet-monocyte aggregates form which express tissue factor. Abbreviations: ACE2 R, angiotensin converting enzyme-2 receptor; aCL, anticardiolipin; aPL, antiphospholipid antibody; β2GPI, beta-2 glycoprotein-I; eNOS, endothelial nitric oxide synthase; NO, nitric oxide; MAC, membrane activation complex; MP, microparticle; NETosis, neutrophil extracellular trap formation/release; ROS, reactive oxygen species; TF, tissue factor.

Endothelial-derived nitric oxide (NO) produced by endothelial NO synthase is required for normal endothelial function. Binding of β2GPI:aPL on endothelial cells disrupts NO production, leading to increased oxidative stress, and activation of endothelial cells, neutrophils and platelets with formation of neutrophil extracellular traps (NETS) and tissue factor rich microparticles.^11^ Furthermore, platelet-monocyte aggregates form, which express tissue factor and activate coagulation.^8,9,12^ Other postulated mechanisms for APS include aPL-induced resistance to activated protein C and downregulation of tissue factor pathway inhibitor.^9^

## Evolution of Antiphospholipid Syndrome Diagnostic Criteria

**Figure 2.**
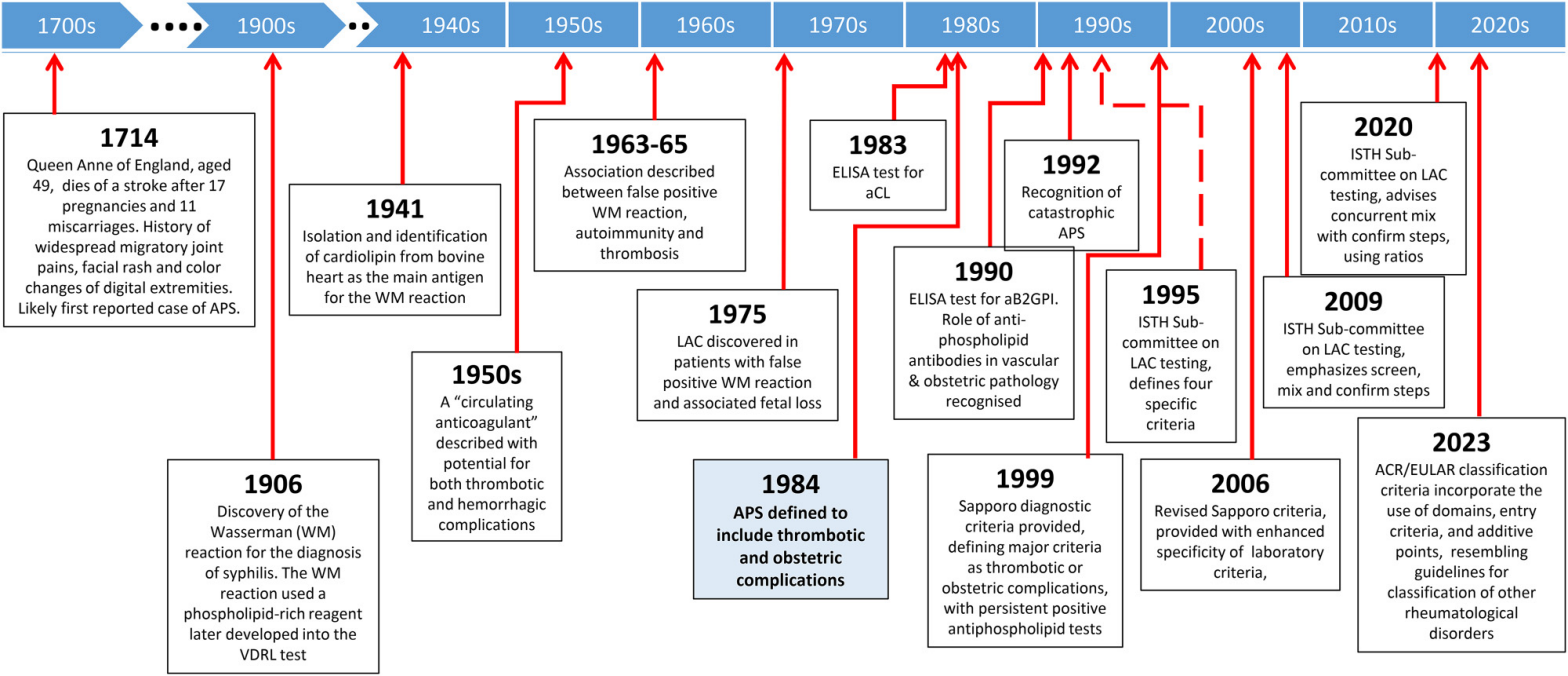
Historical milestones in the recognition and diagnosis of antiphospholipid syndrome.^5,13–18^

Figure 2 provides a timeline overview denoting historical milestones in the recognition and diagnosis of antiphospholipid syndrome. Table 1 compares and summarizes the evolution of diagnostic and classification criteria for APS over the last 25 years. The first diagnostic criteria for definite APS, the Sapporo Criteria, were developed in 1999, and included thrombotic and obstetric complications in the setting of persistent positive laboratory tests at least 6 weeks apart.^13^ In 2006, through an international consensus, an update of the 1999 Sapporo Criteria was issued.^14^ The revised criteria included the addition of a-β2GPI to the antibody test criteria, specified high titers of antibodies, and extended the requirement for persistent test positivity from 6 to 12 weeks to improve specificity. Features associated with APS but not included in the 2006 criteria included valvular heart disease, livedo reticularis, thrombocytopenia, nephropathy and neurological manifestations, including visual complications. In 2023, the American College of Rheumatology/European Alliance of Associations for Rheumatology (ACR/EULAR) provided major revisions to the Sapporo Criteria and algorithms.^15^ ACR/EULAR proposed a new point- and domain-based scoring system which included weighting criteria and a minimum requirement of at least 3 points from the clinical domains, and at least 3 points from the laboratory domains. At least one documented clinical criterion and one qualifying laboratory test performed within the previous 3 years were required as “entry criteria” for APS diagnosis. The recent ACR/EULAR criteria enhanced diagnostic sensitivity at the expense of diagnostic specificity, risking over-diagnosis and potential for “indeterminate” diagnoses.^14,15^ Nonetheless, the use of “entry criteria” encourages clinicians to consider the APS diagnosis in the absence of specified Sapporo Criteria.^13^

Dots indicate significant time lapses (1700s to 1900s and 1900s to 1940s).

Abbreviations: APS, antiphospholipid syndrome; SLE, systemic lupus erythematosus; WM, wassermann; VDRL, venereal disease research laboratory test; LAC, lupus anticoagulant; ELISA, enzyme linked immunoassay; aCL, anti-cardiolipin antibodies**; **a-B2GPI: anti-beta 2 glycoprotein I antibodies; ISTH, international society on thrombosis and hemostasis; ACR, American college of rheumatology; EULAR, European alliance of associations for rheumatology.

**Table 1.** Diagnostic Criteria for Antiphospholipid Syndrome Classification 1999–2023.^13–15^

	1999 Sapporo Criteria	2006 Revision to Sapporo Criteria	2023 ACR/EULAR Classification
Required for APS diagnosis	≥1 clinical criterion (see below) ≥1 laboratory criterion (see below)	≥1 clinical criterion (see below) ≥1 laboratory criterion (see below)	**Entry criteria** for initial evaluation require the *presence of*: 1 clinical criterion (see below) 1 positive test within 3 years (see below) Classification Criteria to **Diagnostic criteria** require APS: 3 + points, clinical domains 1–6 3 + points, laboratory domains 7–8
Clinical criteria	**At least one** of: Vascular Arterial or venous thrombosis* Pregnancy morbidity**	**At least one** of required: Vascular Arterial or venous thrombosis* Pregnancy morbidity** (provided clarification on establishing the diagnoses of pre-eclampsia and placental insufficiency)	**At least one** symptom/clinical domain (D): Domains D1-2: Macrovascular thrombosis* (1–4pts) Arterial or venous thromboses Domain D3: Microvascular (2- vs 5pts) This includes skin, renal, and cardiac manifestations from 2006 criteria Added pulmonary/adrenal and adrenal hemorrhage added Domain D4: Obstetric** (1–4pts) Domain D5: Heart Valve Complications (2- vs 4pts) Domain D6: Thrombocytopenia (2pts)
Relevance of non-diagnostic clinical manifestations	Mentioned and recognized as “Other Features of APS”: Thrombocytopenia Hemolytic anemia Transverse myelitis Livedo reticularis Cardiac valve disease Multiple sclerosis-like syndrome Migraine	Categorized and described, but not formally included formalized in the revised criteria due to insufficient data: Cardiac manifestations Neurological manifestations Skin manifestations Renal manifestations Thrombocytopenia	Except for “non-criteria manifestations” (not related to cerebrovascular events), most other clinical non-criteria manifestations are validated and incorporated into clinical domains as entry and classification criteria are included in the classification. (see above)
Laboratory criteria	**Detectable lupus anticoagulant**: Prolongation of ≥ 1 phospholipid- based coagulation test Failure to correct with mixing studies Corrects with addition of phospholipids Exclude other inhibitors **Positive serology**: aCL, in medium or high titer, IgG or IgM	**Detectable lupus anticoagulant**: No significant changes from 1999 criteria Reference to ISTH scientific subcommittee for expert guidance **Positive serology**: aCL, > 40 units or >99^th^ %tile medium or high titer, IgG or IgM a-B2GPI, > 99^th^ %tile, IgG or IgM	**At least one** laboratory domain (D): Domain D7: Lupus anticoagulant ttesting Single test positive (1pt) Persistent test positive (5pts) positive (5pts) Domain D8: Serology (of aCL, and **a-B2GPI**) IgM only, medium to high titer (1pt) IgG, moderate medium titer (4pts) IgG, high titer, either (5pts) or both (5–7pts)
Criteria Interval to demonstrate for persistent positivity	At least ≥ 6 weeks apart	Between 12 weeks to 5 years apart for APS diagnosis	Between 12 weeks -to to 3 years apart to classify as APS
Sensitivity & specificity for APS detection	Not reported^13^***	86% sensitivity^14^ 99% specificity^14^	99% sensitivity^15^ 84% specificity^15^

Abbreviations: APS, antiphospholipid syndrome; ACR, American College of Rheumatology; EULAR, European Alliance of Associations for Rheumatology; aCL, anti-cardiolipin antibodies; a-B2GPI, Anti-Beta 2 Glycoprotein I Antibodies; pt/pts, point(s).

*The 1999 and 2006 criteria^13,14^ recommended risk-stratifying by pre-existing thrombophilia or cardiovascular disease risks, which are addressed in the 2023 criteria^15^ by separate weighted points (pts). **Pregnancy morbidity and obstetric manifestations include fetal loss with normal morphology or karyotype beyond the 10^th^ week, one or more premature births before 34^th^ week gestation, or three or more unexplained consecutive spontaneous abortions before 10^th^ week.

## Laboratory Testing

**Figure 3.**
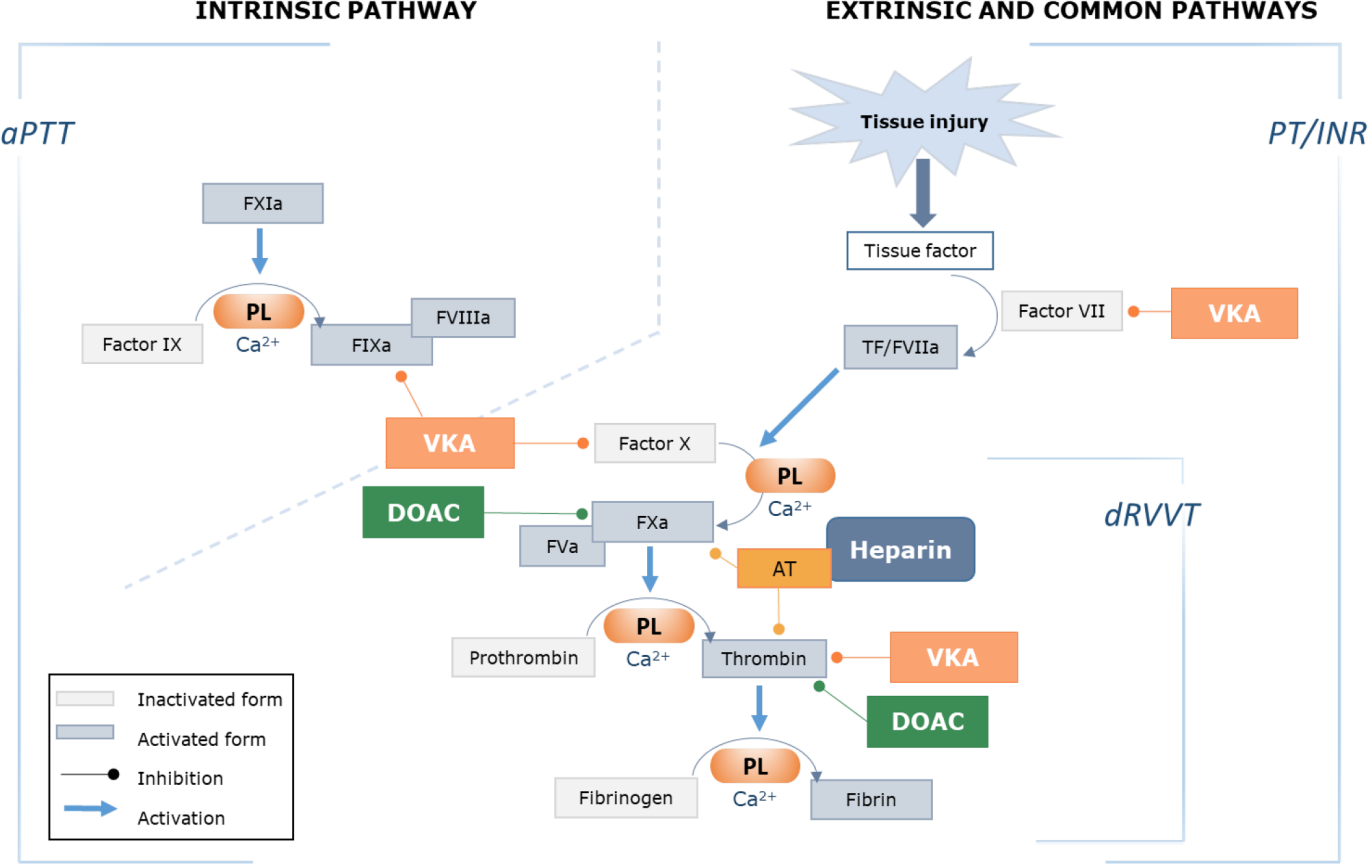
The coagulation cascade, coagulation tests and sites of action of anticoagulant therapy.^17,20–22^

APLs may manifest as LACs using functional clot-based coagulation assays, or be detected immunologically as aCL and/or aB2GPI antibodies in the serum. According to Sapporo Criteria, in the setting of one or more positive clinical criteria, only one laboratory test (LAC, aCL and/or aB2GPI) needs to be persistently positive at least 12 weeks apart for an APS diagnosis (Table 1).^16^ LACs are diagnosed by three basic principles: prolongation of a phospholipid-dependent clotting test, evidence of inhibition demonstrated by mixing studies, and evidence of phospholipid dependence (shortening of the prolonged clotting time with the addition of excess phospholipid).^16,19^ To improve sensitivity and specificity, two clot-based assays using different principles must be used. The dilute Russell viper venom time (dRVVT) and a sensitive activated partial thromboplastin time (aPTT) are the two recommended LAC screening tests.^17^ The dRVVT is based on factor X activation and the aPTT on contact factor activation (Figure 3). There are variations in LAC testing between laboratories and in the use of mixing studies, instrumentation, reagents and calculations. Additional coagulation tests may be used where there is a strong suspicion of LAC with inconclusive DRVVT and aPTT results, or possible assay interference from oral anticoagulants. These additional tests include (but not limited to) the dilute prothrombin time and the kaolin clotting time with platelet neutralization.^20^ LAC testing should be avoided during anticoagulant therapy, particularly with direct oral anticoagulants (DOACs) and vitamin K inhibitors, as well as during the acute thrombotic phase or during pregnancy due to elevated coagulation proteins.^17^

the coagulation screening tests including tests for the lupus anticoagulant (aPTT, DRVVT) are included. Vitamin K antagonists decrease production of factors II, VII, IX, X, proteins C and S (not shown). Heparin binds endogenous antithrombin, potentiating its activity against factors Xa and IIa. DOACs directly inhibit individual activated factors: dabigatran inhibits factor IIa, rivaroxaban, apixaban, and edoxaban inhibit factor Xa. Abbreviations: aPTT, activated partial thromboplastin time; AT, antithrombin; DOAC, direct oral anticoagulant; dRVVT, dilute Russell's viper venom time; INR, international normalized ratio; PL, phospholipid; PT, prothrombin time; VKA, vitamin K antagonist.

The Sapporo Criteria include detection of aCL IgG/IgM antibodies in medium to high titer, and/or a-β2GPI IgG/IgM levels above the 99^th^ centile. Assays used for aCL and a-β2GPI testing including enzyme linked immunosorbent assays (ELISAs) and automated chemiluminescent assays. A challenge is that lack of universal calibrators for these antibody tests leads to inter-assay variation. Follow up testing is recommended in the laboratory that performed the initial tests and using the same testing platform.^14,23^

## Catastrophic Antiphospholipid Syndrome

CAPS is a life-threatening form of APS and is characterized by the rapid onset of widespread thrombotic events affecting three or more organ systems. CAPS is generally induced by a precipitating acute event such as infection, surgery or malignancy.^24,25^ Classification criteria for CAPS were proposed in 2002 at the 10th International Congress on aPL, however, the diagnosis of CAPS remains challenging in real-world settings which require an understanding of both the clinical and laboratory features of this rare disorder.^26^ A confirmed CAPS diagnosis requires involvement of ≥ 3 organ systems, development of clinical manifestations in less than a week, demonstrating small vessel occlusion on histological sections and positive laboratory tests.^27,28^ The pathogenesis is poorly understood, with possible factors including the antibody profile, titers and avidity, and underlying genetic, physiological and environmental factors.^25,29,30^

Due to the rarity of CAPS, and the lack of randomized trials to validate existing diagnostic criteria, recommendations for diagnosis and treatment rely largely on expert opinion.^24^ Approximately half of CAPS patients have a history of APS, and in the remainder it is the first manifestation.^25,31,32^ A high level of suspicion for CAPS is needed for any patient with rapid-onset thrombotic events, progressive multi-organ failure and rapid clinical deterioration.^31^ The 2002 CAPS Classification Criteria categorized patients as “definite” or “probable” CAPS, based on the number of organ systems involved, the time of onset, the presence or absence of histological evidence and the laboratory confirmation of aPLs.^33^ A confirmed diagnosis of “definite” CAPS hinges on persistent aPL however this is not possible in the 50% of cases with no history of APS.^25^ Furthermore, negative aPL profiles at the time of CAPS clinical presentation have been reported.^34^ The McMaster RARE-best practice clinical guidelines recommend using the existing APS classification criteria to diagnose CAPS, emphasizing aPL positivity, albeit with the caution of potential false negatives. Tissue biopsy offers highly specific evidence of CAPS, but may not always be attainable in time.^35^ In addition, comparative data on the risks and benefits of biopsy are not available, perhaps due to the rarity of CAPS.^36^

The differential diagnosis for CAPS with thrombocytopenia and thrombosis in multiple sites includes heparin-induced thrombotic thrombocytopenia (HITT), thrombotic thrombocytopenic purpura (TTP), disseminated intravascular coagulation, scleroderma renal crisis, small vessel vasculitis, and hemolysis, elevated liver enzymes and low platelets (HELLP) syndrome.^24,25^ Some of these entities have validated predictive scoring systems, typical laboratory and clinical findings and diagnostic laboratory tests which would confirm the alternative diagnosis. For instance, a high 4Ts score, the presence of anti-platelet factor 4 antibodies, positive functional testing and a temporal association with heparin supports a diagnosis of HITT, and a severely low ADAMTS13 levels and anti-ADAMTS13 antibodies support a diagnosis of TTP. Importantly, transient aPL, usually aCL, may be detected in any of these conditions.^25^ From an international registry of 522 patients, organ systems most commonly involved in CAPS include renal (73%), pulmonary (60%), central nervous system (56%), cardiac (50%), skin,^37^ hepatic (39%), and peripheral vasculature (37%) involvement.^38^ Common laboratory findings apart from positive aPL tests, include thrombocytopenia in 65% of cases and schistocytes/red cell fragments on the blood smear in 21.7% of cases.^25^

## Lupus Anticoagulant Testing During Anticoagulant Therapy

Lupus anticoagulant and other thrombophilia tests should only be performed where results will alter patient management. For patients on therapeutic anticoagulation, false positive or negative LAC tests may occur,^21^ and therefore LAC testing on anticoagulation therapy is not advised.^17^ Testing should also be avoided in the acute thrombotic phase due to high levels of factor VIII potentially leading to false negative LAC results.^39^ Figure 3 depicts the effects of anticoagulant therapies on the coagulation cascade.

### Lupus Anticoagulant Testing During Warfarin Therapy

The ISTH 2009 guidelines advocate LAC testing only if the INR is <1.5,^18^ however in the 2020 International Society of Thrombosis and Hemostasis (ISTH) guidelines (ISTH 2020) an INR cut-off is not provided. Instead, it is generally advised that LAC testing should be deferred until VKA therapy has been safely discontinued for at least 3 weeks. Alternatively, if essential for clinical management, LAC testing may be performed after three months of adequate VKA therapy by switching temporarily from VKA to therapeutic doses of low molecular weight heparin (LMWH). There is no specific agent available to counteract the VKA effect *in vitro*.^21^ Dilutions of test plasma using 1:1 mix of normal pooled plasma were suggested in the 2009 ISTH Guidelines to correct the VKA effect on factors II, VII, IX and X.^18^ However, this practice fell out of favor in the 2020 updated ISTH guidelines due to laboratory variability in the mixing step.^17^

### Lupus Anticoagulant Testing During Heparin Therapy

Many LAC assays incorporate heparin neutralizing agents such as heparinase, protamine, or polybrene which are generally effective in neutralizing LMWH and unfractionated heparin (UFH) at doses < 1.0 U/mL. However, the effectivity of the neutralizers varies depending on the specific assay and type/brand of heparin used.^39^ For example, a recent study demonstrated that enoxaparin only affected LAC testing at supra-therapeutic anti-Xa levels.^40^ Samples for LAC testing should be collected as close as possible to the next dose of LMWH.^17,39^ For both LMWH and UFH, the ISTH recommends verifying the level of heparin that can be neutralized by reagents claiming to contain heparin neutralizers.^17^

### Lupus Anticoagulant Testing During Direct Oral Anticoagulant Therapy

The dRVVT is particularly susceptible to interference by DOACs, therefore LAC testing should preferably be avoided in this setting.^41,42^ If essential, LAC testing may be conducted at least 48 h after the last DOAC dose, or longer in patients with renal impairment, and DOAC levels should be measured prior to LAC testing.^17,39^ I*n vitro* DOAC neutralizers such as DOAC-Stop^TM^ and DOAC-Remove^TM^ are available, however require further validation.^39,43^ Table 2 provides a summary of the effects of anticoagulants on coagulation tests and proposes strategies for LAC testing in these patients.^17,21,39,43–48^

**Table 2.** Anticoagulant Drugs and Their Effect on Lupus Anticoagulant Testing.^17,21,39,43–48^

Anticoagulant Class	Molecular Targets and Drug Characteristics	Drug Effect on Phospholipid Dependent Coagulation Tests	Summary of Mitigating Strategies
VKA	Prevents gamma-carboxylation of glutamic acid residues on the N-terminus of vitamin K dependent clotting factors Half-life: 36–42 h PK: 95%–100% bioavailability PD: Hepatic metabolism; requires heparin bridging until INR stable. Monitoring: INR	aPTT: ↑ (corrects with mixing studies, unless aPL or heparin interference) dRVVT: ↑↑ (mix step corrects a weak aPL) INR: ↑↑↑ (very rarely prolonged by aPL)	Defer testing until: VKA safely discontinued and INR has normalized, or VKA therapy switched to LMWH (not in first 3 months of treatment)
UFH	Binds to and increases activity of antithrombin 1000-fold Anti-FXa and anti-prothrombin activity. Half-life: 45–60 min PK: Complex mixed-order kinetics PD: Clearance via RES (renal clearance is dose-dependent and saturable) Monitoring: aPTT	aPTT: ↑ to ↑↑↑ (dose-dependent; thrombin time prolonged) dRVVT: ↑ if UFH dose exceeds heparin neutralizer^a^ (false positive screen and mix results)	Use heparin neutralizers Verify level of UFH neutralized before interpretation
LMWH	Binds to and increases activity of antithrombin 1000-fold Mostly Anti-FXa activity Half-life: 240 min PK: First-order kinetics with a clear dose-response relationship PD: Renal clearance (not dose-dependent, non-saturable and linear). Monitoring: not required routinely^b^	aPTT: typically, unaffected^a^ dRVVT: May prolong^a^	Collect samples ≥ 12 h after last dose (longer if ↓CrCl) Depending on LMWH brand, use reagents with heparin neutralizer Verify level of LMWH neutralized before interpretation
DOACs	Direct anti-prothrombin: dabigatran Direct anti-FXa: Rivaroxaban, apixaban, edoxaban Half-life: 9–17 h Metabolism and clearance: vary^c^ Monitoring: not routinely required^b^	aPTT: ↑ to ↑↑ (depending on DOAC) dRVVT: ↑↑ to ↑↑↑ (depending on DOAC)	Test at least 48 h after last dose (longer if ↓CrCl) Check DOAC levels prior to testing Consider a DOAC neutralizer

Abbreviations: aPTT, activated partial thromboplastin time; CrCl, creatinine clearance; DOACs, direct oral anticoagulants; dRVVT, dilute Russel's viper venom time; FXa, activated factor X; INR, international normalized ratio; LA, lupus anticoagulant; LMW, low molecular weight heparin; PK, pharmacokinetics; PD, pharmacodynamics; RES, reticulo-endothelial system; VKA, vitamin K antagonist; UFH, unfractionated heparin.

a Many dRVVT reagents contain a heparin neutralizer able to quench therapeutic doses of UFH and LMWH up to 1 U/mL^39^

b Though routine monitoring is typically not required, in patients with additional risk factors monitoring of drug levels or drug activity may be beneficial^48^

c Dabigatran is primarily cleared renally, while rivaroxaban, apixaban and edoxaban are cleared both renally and via hepatic metabolism^45^

## Confounders in Laboratory Testing

Despite updated ISTH testing recommendations, the practice of aPL and LAC testing remains heterogeneous and variable across individual laboratories, thus creating barriers to standardization of testing. We review and discuss the following sub-topics related to testing and analysis for aPLs.

### Lupus Anticoagulant Test Interpretation

Studies of external quality control surveys have demonstrated wide variability in the detection and interpretation of LACs, which may be due to differences in reagents, sensitivity of reagents to LAC, and variability of practices in interpretation.^37,49^ Nonetheless, a recent international external quality assessment program with approximately 120 participants demonstrated 98% interlaboratory consensus in the correct identification of LAC; in total there were two instances of analytical errors, three instances of interpretation errors, and four instances where the reasons for error were unclear but may be attributed to aPTT-based methods.^49^

The ISTH 2020 Guidelines added emphasis on the use of normalized clotting times for all procedures (screening, confirm, and mixing steps), expressed as ratios, which are calculated as follows^17^:

$$\frac{\mathrm{Numerator}:\mathrm{clotting}\;\mathrm{time},\;\mathrm{patient}/\mathrm{test}\;\mathrm{plasma}}{\mathrm{Denominator}:\mathrm{clotting}\;\mathrm{time},\;\mathrm{normalized}\;\mathrm{value}(e.g.\;\mathrm{normal}\;\mathrm{pooled}\;\mathrm{plasma})}$$

For calculating the normalized ratio, the ISTH 2020 guidelines recommend using daily pooled plasma (NPP) rather than a fixed value, such as the average of the reference range, to account for daily variations.^17^ However, using daily NPP can be challenging due to standardization issues and the risk of operator error.^16^ A recent study with 40 clinical samples and a pool of 50 normal donors found no significant differences in LAC interpretations between using daily NPP and a fixed value for calculations.^50^ Nevertheless, the study confirmed that, as per ISTH recommendations, using normalized clotting times (expressed as ratios) provides more accurate LAC detection compared to using clotting times alone.^50^ However, the actual impact of adopting different variations of normalized ratios on patient care, in particular with diagnosing APS and SLE risk stratification, are yet to be ascertained.

### Caveats of the Mixing Study and Mixing Steps

LACs typically act as inhibitors when performing the aPTT mixing test with NPP in a 1:1 ratio, and the prolonged test fails to correct. However, in the case of a weak LAC, the aPTT may correct into the reference range with mixing, leading to misinterpretation as a factor deficiency.^51^ In the ISTH 2009 guidelines, a prolonged DRVVT or aPTT normalized screen time should be followed by a 1:1 mixing test with NPP. If the screen is prolonged, but the screen 1:1 mix corrects into the normal range, the interpretation should be negative, and a factor deficiency should be considered.^18^ In this scenario, a low strength LAC may be missed.

As highlighted by the ISTH 2020 Guidelines on LAC testing and interpretation, a mixing step is required if the screening LAC assay is prolonged (DRVVT or aPTT).^17,18^ However, the ISTH 2020 guidelines also advise on performing the confirm test [eg DRVVT confirm or Silica Clotting Time (SCT)] since normalized screening times are prolonged. If the confirm test corrects, test ratios (or percent correction) should be calculated using the *unmixed* screen and confirm ratios. If the confirm test remains elevated, then the 1:1 mix is also performed on the confirm, and the test ratio (or percent correction) should be calculated using *mixed* screen and confirm ratios.

**Figure 4.**
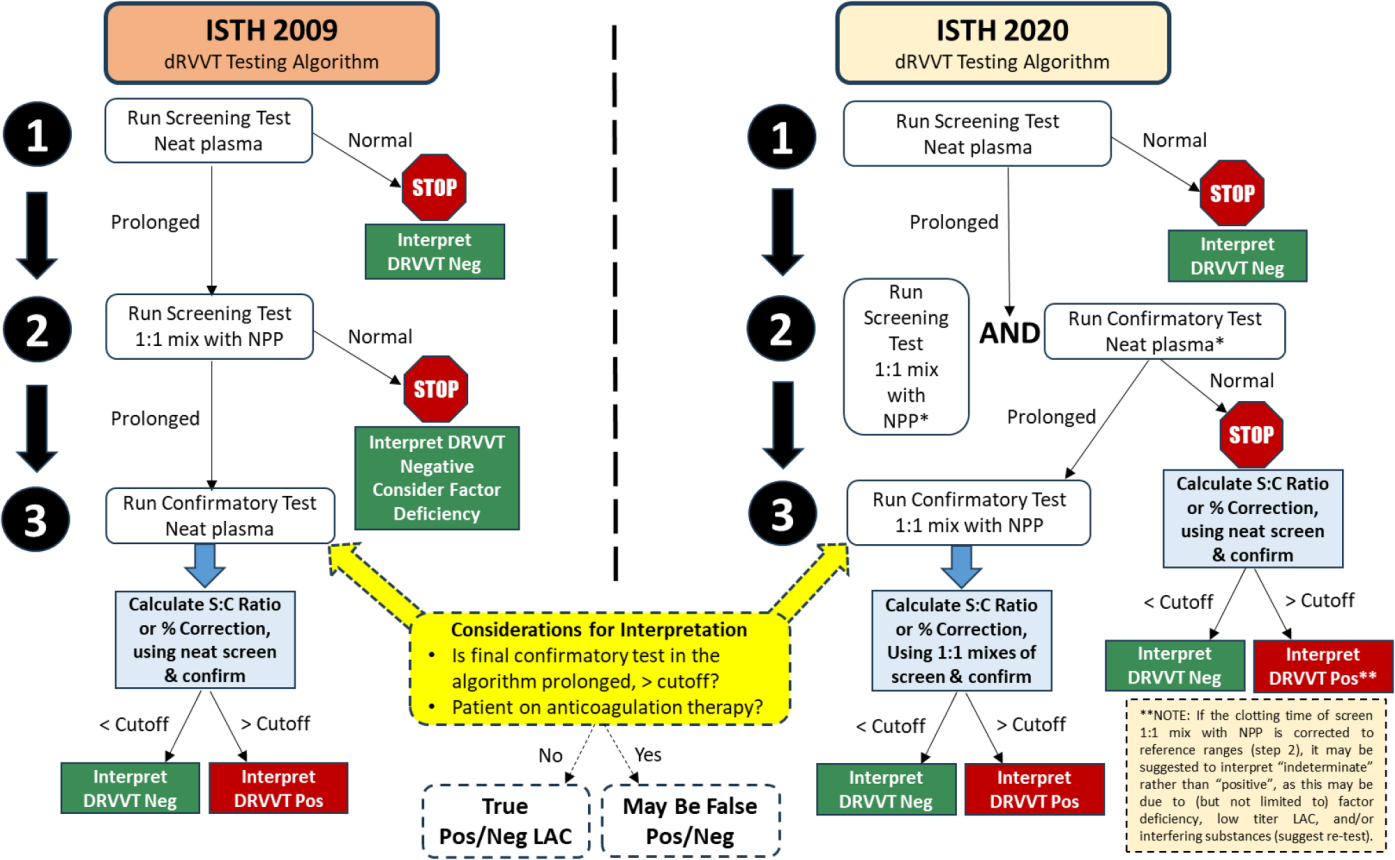
Comparison of 2009 and 2020 ISTH lupus anticoagulant testing algorithms.^18,39^

Using DRVVT as an example, Figure 4 compares the ISTH 2020 and 2009 guidelines to the use of screen, confirm, and mixing step(s), with interpretation options. The ISTH 2020 guidelines improve the detection of weak LACs, reduce incorrect conclusions of factor deficiencies, and facilitate further appropriate investigation. Use of clinical information to aid interpretation may be helpful or necessary when the pattern of testing appears “indeterminate” (mildly prolonged screen test, corrected screen 1:1 mix test, corrected confirm test, elevated screen : confirm ratio above cutoff indicating a “positive” result, and a normal screen : confirm mix ratio indicating a “negative” result). The clinical significance of such “indeterminate” result patterns should be further investigated, as no standard guideline is provided on these results should be interpreted.

this illustration uses the dRVVT assay as an example. Neat plasma represents undiluted sample plasma. *Whereas a Confirmatory test is only performed if Screen with 1:1 mix with NPP show elevation above the reference range per ISTH 2009 guidelines, in the ISTH 2020 guidelines the Confirmatory test is also performed if screening test suggests LA, irrespective of mixing test result. Abbreviations: ISTH, International Society of Thrombosis and Haemostasis; NPP, normal plasma pooled from at least 40 donors; LAC, lupus anticoagulant; Neg, negative; Pos, positive; S:C, screen to confirm calculated using normalized clotting ratios.

### Seronegative Antiphospholipid Syndrome

The entity seronegative APS (SN-APS) has been introduced for patients with clinical manifestations of APS with negative standard laboratory tests.^52^ Several studies have explored the presence of other aPL which do not form part of the current diagnostic criteria. These include anti-phosphatidylserine/prothrombin antibodies, antiphosphatidylethanolamine antibodies, anti-annexin A5, IgA isotype a-Β2GPI, antiphosphatidylinisitol, antiphosphatidic acid, and antivimentin/cardiolipin antibodies. One study including 175 consecutive patients with SN-APS, demonstrated that a third of cases showed reactivity to non-criteria aPL.^53^ Although there is evidence supporting the role of testing for alternative aPLs in suspected SN-APS cases, these tests are not routinely available.^54–56^ Furthermore, it is unclear what their prevalence is in healthy controls. To ascertain the clinical significance of non-conventional aPLs, future studies should incorporate healthy controls to better assess their prevalence and clinical significance.^54^

### “False Negative” Antiphospholipid Antibodies

In addition to advising against testing during therapeutic anticoagulation, The 2020 ISTH guidelines also caution LAC testing following acute thrombotic events, since raised acute phase reactants such as factor VIII and CRP may yield false positive or negative LAC results.^17^ Previous studies have described the temporal disappearance of aPL during a thrombotic event, which may lead to a missed diagnosis.^57,58^ In a prospective study of SLE patients with recent thrombotic events that studied aCL and LACs, aPL previously positive became negative in half the cohort (aCL IgG, by 41%; IgM, by 51%; IgA, by 50%; LAC, by 20%). The majority of those who “lost” aCL IgG and LAC positivity following thrombosis, regained positivity.^57^ Other factors which may result in a false negative aPL include pre-analytical factors such as hemolysis, renal loss in nephrotic syndrome, pregnancy, and use of immunosuppressive drugs such as corticosteroids and rituximab.^16,59^

## Management of the Antiphospholipid Syndrome

Universal evidence based therapeutic guidelines are scarce due to the broad clinical spectrum and rarity of APS which precludes well-designed prospective studies. Long term anticoagulation therapy is needed for aPL-associated unprovoked/recurrent/extensive venous thrombosis,^60,61^ with recent studies supporting moderate intensity dose warfarin (INR 2–3) rather than high intensity dose warfarin (INR 3.1-4).^4^ Anticoagulation therapy and treatment should be individualized to ensure that the benefit for each patient outweighs the risk of bleeding. EULAR guidelines suggest low dose aspirin for primary prophylaxis in high-risk patients who do not meet clinical diagnostic criteria for APS.^4,15^ Treatment and preventive options for obstetric APS include combined low dose aspirin and heparin, low dose prednisolone, intravenous immunoglobulin (IVIG) and hydroxychloroquine.^62,63^ Due to the high mortality of CAPS, empiric treatment is advised. Treatment options include glucocorticoids, therapeutic heparin, plasma exchange, IVIG, rituximab and eculizumab. Underlying possible precipitants of CAPS also need to be aggressively treated.^64^ The combined regimen of immunosuppression, plasma exchange, and therapeutic anticoagulation significantly improved survival and recovery of CAPS in one study (62% vs 23%, p = .014).^64^ DOAC therapy should be avoided in triple positive APS, with combined LAC, aCL and aB2GPI positivity, due to high risk of recurrent thrombosis and warfarin is favored in this setting.^65–67^ Vitamin K antagonists and DOACs are contraindicated during pregnancy.^68^

## Conclusion

APS is a complex and challenging autoimmune disorder, and appropriate management requires an understanding of its pathophysiology, diagnostic criteria, and therapies. Despite advances in the development of classification criteria, the diagnosis remains complex due to disease heterogeneity and the variability in laboratory testing. While the Sapporo Criteria for APS diagnosis have been the gold standard for two decades, the 2023 ACR/EULAR Criteria aimed to enhance diagnostic specificity, however at the cost of potential over-diagnosis. Prompt and appropriate treatment is needed to improve patient outcomes, particularly in CAPS. As our understanding of the pathophysiology and epidemiology improves, together with refined diagnostic approaches, therapeutic advances will be crucial in improving clinical outcomes in these patients.

## Key Points

- The latest ACR/EULAR classification guidelines are comprehensive and improves diagnostic sensitivity but may require additional clinical correlation due to decreased specificity, compared to the Sapporo criteria.
- Catastrophic APS remains a rare entity that is clinically challenging to diagnose and manage, with a high mortality rate.
- Mitigation strategies for APS testing should consider pre-test variables such as anticoagulation therapy or acute phases of illness which may render false positive or negative results.
- Despite the most recent ISTH guidelines on laboratory testing for APS, confounders to laboratory testing and result interpretation exist; their significance on patient care is yet to be ascertained.

## References

[1] Mezhov V Segan JD Tran H Cicuttini FM . Antiphospholipid syndrome: A clinical review. Med J Aust. 2019;211(4):184–188. 31271468 10.5694/mja2.50262

[2] Xourgia E Tektonidou MG . An update on antiphospholipid syndrome. Curr Rheumatol Rep. 2022;23(12):84. 34985625 10.1007/s11926-021-01051-5

[3] Dabit JY Valenzuela-Almada MO Vallejo-Ramos S Duarte-Garcia A . Epidemiology of antiphospholipid syndrome in the general population. Curr Rheumatol Rep. 2022;23(12):85. 34985614 10.1007/s11926-021-01038-2 PMC8727975

[4] Sammaritano LR . Antiphospholipid syndrome. Best Pract Res Clin Rheumatol. 2020;34(1):101463. 31866276 10.1016/j.berh.2019.101463

[5] Tincani A Fontana G Mackworth-Young C . The history of antiphospholipid syndrome. Reumatismo. 2023;74(4). doi:10.4081/reumatismo.2022.1556. 36942979

[6] Chaturvedi S McCrae KR . Diagnosis and management of the antiphospholipid syndrome. Blood Rev. 2017;31(6):406–417. 28784423 10.1016/j.blre.2017.07.006 PMC5714279

[7] Wong RC Favaloro EJ . Clinical features, diagnosis, and management of the antiphospholipid syndrome. Semin Thromb Hemost. 2008;34(3):295–304. 18720311 10.1055/s-0028-1082275

[8] Schreiber K Sciascia S de Groot PG , et al. Antiphospholipid syndrome. Nat Rev Dis Primers. 2018;4(1):17103. 29321641 10.1038/nrdp.2017.103

[9] Arachchillage DRJ Laffan M . Pathogenesis and management of antiphospholipid syndrome. Br J Haematol. 2017;178(2):181–195. 28339096 10.1111/bjh.14632

[10] McDonnell T Wincup C Buchholz I , et al. The role of beta-2-glycoprotein I in health and disease associating structure with function: More than just APS. Blood Rev. 2020;39:100610. doi:10.1016/j.blre.2019.100610. 31471128 PMC7014586

[11] Corban MT Duarte-Garcia A McBane RD Matteson EL Lerman LO Lerman A . Antiphospholipid syndrome: Role of vascular endothelial cells and implications for risk stratification and targeted therapeutics. J Am Coll Cardiol. 2017;69(18):2317–2330. 28473138 10.1016/j.jacc.2017.02.058

[12] Raschi E Borghi MO Tedesco F Meroni PL . Antiphospholipid syndrome pathogenesis in 2023: An update of new mechanisms or just a reconsideration of the old ones? Rheumatology (Oxford). 2024;63(SI):SI4–SI13. 10.1093/rheumatology/kead603 38320591

[13] Wilson WA Gharavi AE Koike T , et al. International consensus statement on preliminary classification criteria for definite antiphospholipid syndrome: Report of an international workshop. Arthritis Rheum. 1999;42(7):1309–1311. 10403256 10.1002/1529-0131(199907)42:7<1309::AID-ANR1>3.0.CO;2-F

[14] Miyakis S Lockshin MD Atsumi T , et al. International consensus statement on an update of the classification criteria for definite antiphospholipid syndrome (APS). J Thromb Haemost. 2006;4(2):295–306. 16420554 10.1111/j.1538-7836.2006.01753.x

[15] Barbhaiya M Zuily S Naden R , et al. The 2023 ACR/EULAR antiphospholipid syndrome classification criteria. Arthritis Rheumatol. 2023;75(10):1687–1702. 37635643 10.1002/art.42624

[16] Brandt JT Triplett DA Alving B Scharrer I . Criteria for the diagnosis of lupus anticoagulants: An update. On behalf of the subcommittee on lupus anticoagulant/antiphospholipid antibody of the scientific and standardisation committee of the ISTH. Thromb Haemost. 1995;74(4):1185–1190. 8560433

[17] Devreese KMJ de Groot PG de Laat B , et al. Guidance from the scientific and standardization committee for lupus anticoagulant/antiphospholipid antibodies of the international society on thrombosis and haemostasis: Update of the guidelines for lupus anticoagulant detection and interpretation. J Thromb Haemost. 2020;18(11):2828–2839. 33462974 10.1111/jth.15047

[18] Pengo V Tripodi A Reber G , et al. Update of the guidelines for lupus anticoagulant detection. Subcommittee on lupus anticoagulant/antiphospholipid antibody of the scientific and standardisation committee of the international society on thrombosis and haemostasis. J Thromb Haemost. 2009;7(10):1737–1740. 19624461 10.1111/j.1538-7836.2009.03555.x

[19] Wisloff F Jacobsen EM Liestol S . Laboratory diagnosis of the antiphospholipid syndrome. Thromb Res. 2002;108(5-6):263–271. 12676184 10.1016/s0049-3848(02)00400-0

[20] Favaloro EJ Pasalic L Selby R . Testing for the lupus anticoagulant: The good, the bad, and the ugly. Res Pract Thromb Haemost. 2024;8(3):102385. 38623474 10.1016/j.rpth.2024.102385 PMC11017341

[21] Favaloro EJ Pasalic L . Lupus anticoagulant testing during anticoagulation, including direct oral anticoagulants. Res Pract Thromb Haemost. 2022;6(2):e12676. 10.1002/rth2.12676 PMC8922544 35316943

[22] Cross B Turner RM Zhang JE Pirmohamed M . Being precise with anticoagulation to reduce adverse drug reactions: Are we there yet? Pharmacogenomics J. 2024;24(2):7. 38443337 10.1038/s41397-024-00329-y PMC10914631

[23] Vandevelde A Devreese KMJ . Laboratory diagnosis of antiphospholipid syndrome: insights and hindrances. J Clin Med. 2022;11(8):2164. 10.3390/jcm11082164 PMC9025581 35456258

[24] Carmine Siniscalchi MB Riva M Meschi M Meschi T Castaldo G Di Micco P . Catastrophic antiphospholipid syndrome: A review. Immuno. 2024;4(1):1–13.

[25] Cervera R Rodriguez-Pinto I Colafrancesco S , et al. 14th International congress on antiphospholipid antibodies task force report on catastrophic antiphospholipid syndrome. Autoimmun Rev. 2014;13(7):699–707. 24657970 10.1016/j.autrev.2014.03.002

[26] Cervera R Rodriguez-Pinto I Espinosa G . The diagnosis and clinical management of the catastrophic antiphospholipid syndrome: A comprehensive review. J Autoimmun. 2018;92:1–11. doi: 10.1016/j.jaut.2018.05.007. 29779928

[27] Cervera R Espinosa G . Update on the catastrophic antiphospholipid syndrome and the “CAPS registry”. Semin Thromb Hemost. 2012;38(4):333–338. 22618528 10.1055/s-0032-1304718

[28] Kazzaz NM McCune WJ Knight JS . Treatment of catastrophic antiphospholipid syndrome. Curr Opin Rheumatol. 2016;28(3):218–227. 26927441 10.1097/BOR.0000000000000269 PMC4958413

[29] Asherson RA . The catastrophic antiphospholipid (Asherson's) syndrome in 2004–a review. Autoimmun Rev. 2005;4(1):48–54. 15652779 10.1016/j.autrev.2004.03.007

[30] Ortega-Hernandez OD Agmon-Levin N Blank M Asherson RA Shoenfeld Y . The physiopathology of the catastrophic antiphospholipid (Asherson's) syndrome: Compelling evidence. J Autoimmun. 2009;32(1):1–6. 19059760 10.1016/j.jaut.2008.10.003

[31] Cervera R Bucciarelli S Plasin MA , et al. Catastrophic antiphospholipid syndrome (CAPS): Descriptive analysis of a series of 280 patients from the “CAPS registry”. J Autoimmun. 2009;32(3-4):240–245. 19324520 10.1016/j.jaut.2009.02.008

[32] Sevim E Zisa D Andrade D , et al. Characteristics of patients with antiphospholipid antibody positivity in the APS ACTION international clinical database and repository. Arthritis Care Res (Hoboken). 2022;74(2):324–335. 32986935 10.1002/acr.24468 PMC10725727

[33] Asherson RA Cervera R de Groot PG , et al. Catastrophic antiphospholipid syndrome: International consensus statement on classification criteria and treatment guidelines. Lupus. 2003;12(7):530–534. 12892393 10.1191/0961203303lu394oa

[34] Nayfe R Uthman I Aoun J Saad Aldin E Merashli M Khamashta MA . Seronegative antiphospholipid syndrome. Rheumatology (Oxford). 2013;52(8):1358–1367. 23502076 10.1093/rheumatology/ket126

[35] Erkan D Espinosa G Cervera R . Catastrophic antiphospholipid syndrome: Updated diagnostic algorithms. Autoimmun Rev. 2010;10(2):74–79. 20696282 10.1016/j.autrev.2010.08.005

[36] Legault K Schunemann H Hillis C , et al. Mcmaster RARE-bestpractices clinical practice guideline on diagnosis and management of the catastrophic antiphospholipid syndrome. J Thromb Haemost. 2018;16(8):1656–1664. 29978552 10.1111/jth.14192

[37] Reber G Meijer P . In ECAT veritas? Lupus. 2012;21(7):722–724. 22635212 10.1177/0961203312446389

[38] Rodriguez-Pinto I Moitinho M Santacreu I , et al. Catastrophic antiphospholipid syndrome (CAPS): Descriptive analysis of 500 patients from the international CAPS registry. Autoimmun Rev. 2016;15(12):1120–1124. 27639837 10.1016/j.autrev.2016.09.010

[39] Tripodi A Cohen H Devreese KMJ . Lupus anticoagulant detection in anticoagulated patients. Guidance from the scientific and standardization committee for lupus anticoagulant/antiphospholipid antibodies of the international society on thrombosis and haemostasis. J Thromb Haemost. 2020;18(7):1569–1575. 32619349 10.1111/jth.14846

[40] De Kesel PMM Devreese KMJ . The effect of unfractionated heparin, enoxaparin, and danaparoid on lupus anticoagulant testing: Can activated carbon eliminate false-positive results? Res Pract Thromb Haemost. 2020;4(1):161–168. 31989098 10.1002/rth2.12264 PMC6971310

[41] Favaloro EJ Mohammed S Curnow J Pasalic L . Laboratory testing for lupus anticoagulant (LA) in patients taking direct oral anticoagulants (DOACs): Potential for false positives and false negatives. Pathology. 2019;51(3):292–300. 30665674 10.1016/j.pathol.2018.11.008

[42] Hoxha A Banzato A Ruffatti A Pengo V . Detection of lupus anticoagulant in the era of direct oral anticoagulants. Autoimmun Rev. 2017;16(2):173–178. 27988438 10.1016/j.autrev.2016.12.010

[43] Arachchillage DRJ Gomez K Alikhan R , et al. Addendum to British society for haematology guidelines on investigation and management of antiphospholipid syndrome, 2012 (Br. J. Haematol. 2012; 157: 47-58): Use of direct acting oral anticoagulants. Br J Haematol. 2020;189(2):212–215. 31943138 10.1111/bjh.16308

[44] Alban S . Adverse effects of heparin. Handb Exp Pharmacol. 2012(207):211–263. doi:10.1007/978-3-642-23056-1_10. 22566227

[45] Chen A Stecker E B AW . Direct oral anticoagulant use: A practical guide to common clinical challenges. J Am Heart Assoc. 2020;9(13):e017559. 10.1161/JAHA.120.017559 PMC7670541 32538234

[46] Hirsh J Fuster V Ansell J Halperin JL , American Heart A, American College of Cardiology F. American Heart Association/American College of Cardiology foundation guide to warfarin therapy. Circulation. 2003;107(12):1692–1711. 12668507 10.1161/01.CIR.0000063575.17904.4E

[47] Scaglione F . New oral anticoagulants: Comparative pharmacology with vitamin K antagonists. Clin Pharmacokinet. 2013;52(2):69–82. 23292752 10.1007/s40262-012-0030-9

[48] Qiao J Tran MH . Challenges to laboratory monitoring of direct oral anticoagulants. Clin Appl Thromb Hemost. 2024;30:10760296241241524. doi:10.1177/10760296241241524. 38650302 PMC11036927

[49] Favaloro EJ Dean E Arunachalam S . Variable performance of lupus anticoagulant testing: the australasian/asia-pacific experience. Semin Thromb Hemost. 2023;50(8):1103–1113. doi:10.1055/s-0043-1776406. 37967835

[50] Ling LQ Liu CN Huang XB Liao J Jia J Zhou J . Interpretation of clot-based lupus anticoagulant assays-normalizing clotting time against different denominators. Int J Lab Hematol. 2022;44(4):777–784. 35297205 10.1111/ijlh.13830

[51] Chighizola CB Raschi E Banzato A Borghi MO Pengo V Meroni PL . The challenges of lupus anticoagulants. Expert Rev Hematol. 2016;9(4):389–400. 26789237 10.1586/17474086.2016.1140034

[52] Hughes GR Khamashta MA . Seronegative antiphospholipid syndrome. Ann Rheum Dis. 2003;62(12):1127. 14644846 10.1136/ard.2003.006163 PMC1754381

[53] Zohoury N Bertolaccini ML Rodriguez-Garcia JL , et al. Closing the serological gap in the antiphospholipid syndrome: The value of “non-criteria” antiphospholipid antibodies. J Rheumatol. 2017;44(11):1597–1602. 28864642 10.3899/jrheum.170044

[54] Conti F Andreoli L Crisafulli F Mancuso S Truglia S Tektonidou MG . Does seronegative obstetric APS exist? “pro” and “cons”. Autoimmun Rev. 2019;18(12):102407. 31639518 10.1016/j.autrev.2019.102407

[55] Hughes GRV Khamashta MA . Seronegative antiphospholipid syndrome': An update. Lupus. 2019;28(3):273–274. 30691344 10.1177/0961203319826358

[56] Litvinova E Darnige L Kirilovsky A Burnel Y de Luna G Dragon-Durey MA . Prevalence and significance of non-conventional antiphospholipid antibodies in patients with clinical APS criteria. Front Immunol. 2018;9:2971. doi:10.3389/fimmu.2018.02971. 30619328 PMC6302212

[57] Khawaja M Magder L Goldman D Petri MA . Loss of antiphospholipid antibody positivity post-thrombosis in SLE. Lupus Sci Med. 2020;7(1):e000423. doi:10.1136/lupus-2020-000423. PMC7539588 33023978

[58] Miret C Cervera R Reverter JC , et al. Antiphospholipid syndrome without antiphospholipid antibodies at the time of the thrombotic event: Transient ‘seronegative’ antiphospholipid syndrome? Clin Exp Rheumatol. 1997;15(5):541–544. 9307863

[59] Perez-Vazquez ME Cabiedes J Cabral AR Alarcon-Segovia D . Decrease in serum antiphospholipid antibody levels upon development of nephrotic syndrome in patients with systemic lupus erythematosus: Relationship to urinary loss of IgG and other factors. Am J Med. 1992;92(4):357–362. 1558081 10.1016/0002-9343(92)90264-c

[60] Connors JM . Thrombophilia testing and venous thrombosis. N Engl J Med. 2017;377(12):1177–1187. 28930509 10.1056/NEJMra1700365

[61] Kearon C Parpia S Spencer FA , et al. Antiphospholipid antibodies and recurrent thrombosis after a first unprovoked venous thromboembolism. Blood. 2018;131(19):2151–2160. 29490924 10.1182/blood-2017-09-805689 PMC6536697

[62] Depietri L Veropalumbo MR Leone MC Ghirarduzzi A . Antiphospholipid syndrome: state of the art of clinical management. Cardiovasc Drugs Ther. 2023. doi:10.1007/s10557-023-07496-3. 37572208

[63] Farquharson RG Quenby S Greaves M . Antiphospholipid syndrome in pregnancy: A randomized, controlled trial of treatment. Obstet Gynecol. 2002;100(3):408–413. 12220757 10.1016/s0029-7844(02)02165-8

[64] Alkayed K Kottke-Marchant K . Indeterminate lupus anticoagulant results: Prevalence and clinical significance. Korean J Hematol. 2011;46(4):239–243. 22259629 10.5045/kjh.2011.46.4.239 PMC3259515

[65] Pengo V Denas G Zoppellaro G , et al. Rivaroxaban vs warfarin in high-risk patients with antiphospholipid syndrome. Blood. 2018;132(13):1365–1371. 30002145 10.1182/blood-2018-04-848333

[66] Pengo V Ruffatti A Del Ross T , et al. Confirmation of initial antiphospholipid antibody positivity depends on the antiphospholipid antibody profile. J Thromb Haemost. 2013;11(8):1527–1531. 23601766 10.1111/jth.12264

[67] Pengo V Ruffatti A Legnani C , et al. Incidence of a first thromboembolic event in asymptomatic carriers of high-risk antiphospholipid antibody profile: A multicenter prospective study. Blood. 2011;118(17):4714–4718. 21765019 10.1182/blood-2011-03-340232

[68] Lippi G Gosselin R Favaloro EJ . Current and emerging direct oral anticoagulants: State-of-the-art. Semin Thromb Hemost. 2019;45(5):490–501. 31216588 10.1055/s-0039-1692703

